# Patient-Reported Outcome Measures in Routine Clinical Practice: *Practical Guidance for Institutional Review Boards*

**DOI:** 10.1002/eahr.500216

**Published:** 2024

**Authors:** Justin M. Bachmann, Molly A. Shiflet, Julia R. Palacios, Robert W. Turer, Grace H. Wallace, S. Trent Rosenbloom, Todd W. Rice

**Affiliations:** Justin M. Bachmann, MD, MPH, is an assistant professor of medicine and biomedical informatics and medical director for patient-reported outcomes measurement at Vanderbilt University Medical Center. He is also a staff physician and research scientist at the Veterans Affairs Tennessee Valley Healthcare System; Molly A. Shiflet, MPH, is an operations manager for the human research protections program at Vanderbilt University Medical Center; Julia R. Palacios, BS, is a medical student at Saint Louis University School of Medicine; Robert W. Turer, MD, MSE, is an assistant professor of emergency medicine at University of Texas-Southwestern Medical Center; Grace H. Wallace, BS, is a clinical/translational research coordinator at Vanderbilt University Medical Center; S. Trent Rosenbloom, MD, MPH, is a professor of biomedical informatics, medicine, pediatrics, and nursing, and is a vice chair of faculty affairs in the Department of Biomedical Informatics at Vanderbilt University Medical Center. He also directs My Health at Vanderbilt, Vanderbilt’s patient portal; Todd W. Rice, MD, MSc, is a professor of medicine and vice president for clinical trial innovation and operations as well as medical director of the human research protections program at Vanderbilt University Medical Center.

**Keywords:** patient-reported outcome measures, institutional review board, human subjects research, quality improvement, clinical care, patient-centered outcomes research

## Abstract

The use of patient-reported outcome measures (PROMs) is increasingly common in routine clinical practice. As tools to quantify symptoms and health status, PROMs play an important role in focusing health care on outcomes that matter to patients. The uses of PROM data are myriad, ranging from clinical care to survey-based research and quality improvement. Discerning the boundaries between these use cases can be challenging for institutional review boards (IRBs). In this article, we provide a framework for classifying the three primary PROM use cases (clinical care, human subjects research, and quality improvement) and discuss the level of IRB oversight (if any) necessary for each. One of the most important considerations for IRB staff is whether PROMs are being used primarily for clinical care and thus do not constitute human subjects research. We discuss characteristics of PROMs implemented primarily for clinical care, focusing on: data platform; survey location; questionnaire length; patient interface; and clinician interface. We also discuss IRB oversight of projects involving the secondary use of PROM data that were collected during the course of clinical care, which span human subjects research and quality improvement. This framework provides practical guidance for IRB staff as well as clinicians who use PROMs as communication aids in routine clinical practice.

Patient-reported outcomes are reports of health status, quality of life, and symptoms that come directly from the patient.^1^ Patient-reported outcomes are quantified with patient-reported outcome measures (PROMs), psychometrically-validated instruments that transform health status into numerical scores.^2^ In the past, PROMs were largely employed as research tools, used in clinical trials and observational studies to quantify symptoms of interest.^3^ More recently, PROMs have found utility in routine, real-time clinical practice as a way for clinicians to monitor outcomes that matter to patients.^4^ Value-based health care delivery models are increasingly focusing on the use of PROMs as performance measures.^5^ The Centers for Medicare & Medicaid Services (CMS) have added metrics involving the integration of PROMs into electronic health records (EHR) to the Merit-Based Incentive Payment System,^6^ and *U.S. News & World Report* includes metrics related to PROMs in its hospital ranking methodology.^7^ More recently, CMS established total hip and knee arthroplasty patient-reported outcome-based performance measures as part of its Hospital Inpatient Quality Reporting Program.^8^

Given the multiple-use cases for PROMs, institutional review board (IRB) oversight for research projects using these measures (and determinations whether oversight is necessary) can be challenging. Accordingly, this article is primarily intended for IRB staff at medical centers that use these questionnaires during routine clinical care. It is also intended to assist clinicians who implement and use PROMs as clinician-patient communication tools, helping them engage with IRBs when necessary. Lastly, this article will inform researchers who study PROM data, providing context regarding how these measures are operationalized in clinical care.

In this article, we discuss the taxonomy of PROM use cases and present a practical framework for IRB oversight of PROM data. Appropriately classifying PROM use cases assists IRBs in determining the level of oversight and informed consent necessary for research use of PROMs collected during clinical care. This framework is informed by clinical, operational, and research experience from an enterprise-level initiative to integrate PROMs into routine clinical care at a large academic health center. We begin by discussing the primary use cases of PROM data (clinical, human subjects research, and quality improvement [QI]). These use cases determine the level of IRB oversight necessary for PROMs implemented in the clinical setting. We then discuss the secondary use of PROM data collected primarily for clinical care in QI and research endeavors, including the level of informed consent necessary for the research use of PROMs. We provide criteria to assist IRBs in determining whether PROMs were implemented primarily for routine clinical care, as opposed to primarily for research or QI. We then discuss the potential uses of PROM data for QI purposes, with a focus on the characteristics of exempt versus nonexempt PROM-related QI activities. A nuanced understanding of the myriad uses of PROM data is important for IRB staff as well as clinicians and administrators at institutions that seek to better quantify the health status of their patients.

## THE PATIENT-REPORTED DATA SPECTRUM

**Figure 1.**
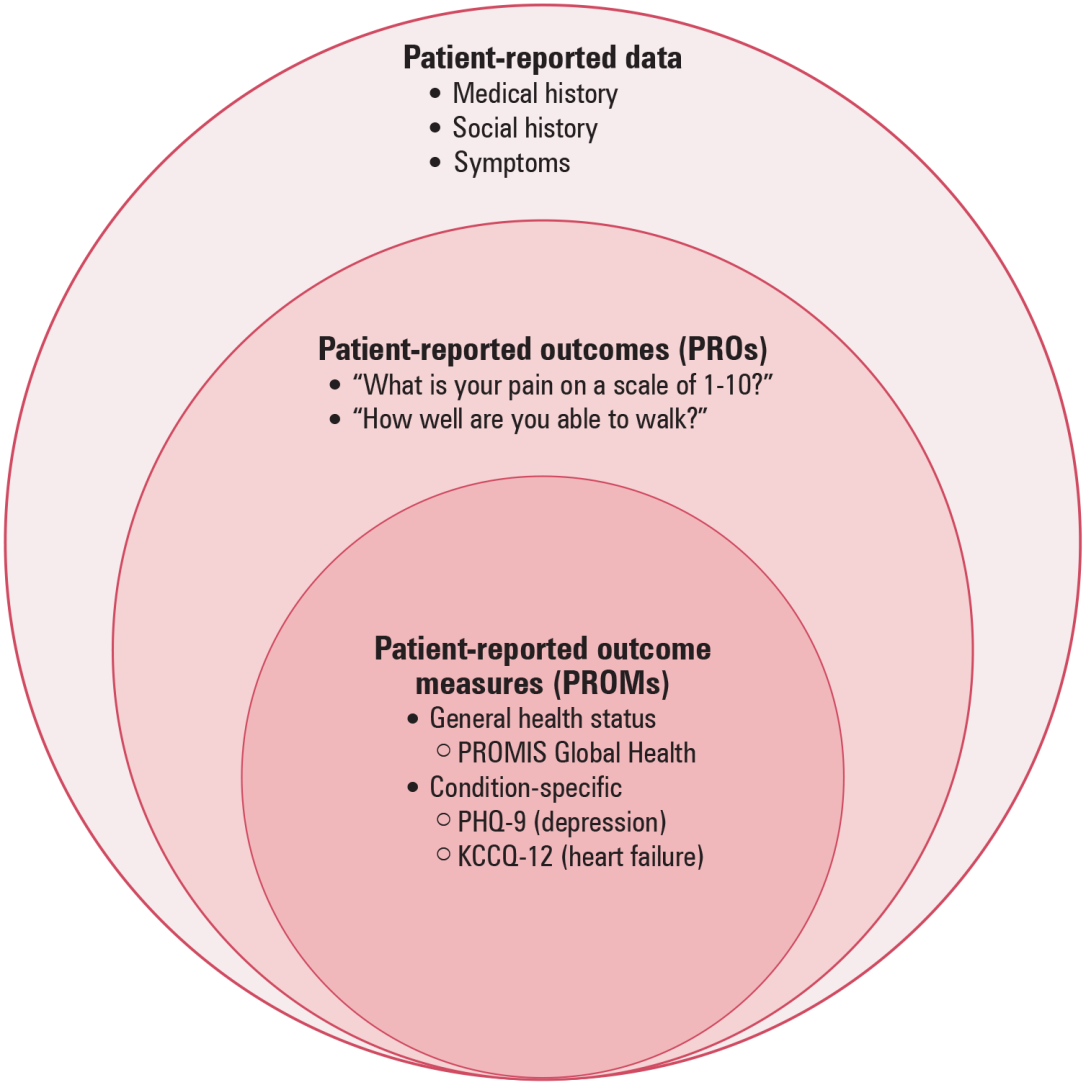
PROs and PROMs as Part of the Patient-Reported Data Spectrum PROMs are a subset of PROs that evaluate patient health status with validated questionnaires. Patient-reported outcome (PRO); Patient-reported outcome measure (PROM); Patient-Reported Outcomes Measurement Information System Global Health (PROMIS Global Health); Patient Health Questionnaire 9 (PHQ-9); Kansas City Cardiomyopathy Questionnaire Short Form (KCCQ-12).

For the purposes of this article, we define patient-reported data as any data reported by the patient without interpretation by the clinician or anyone else (see figure 1). Examples of patient-reported data include medical history, social history, and symptoms. We define patient-reported outcomes (PROs), a subset of patient-reported data, as any report of symptoms or health status that comes directly from the patient.^9^ PROs may be recorded in the form of a score but need not be psychometrically validated. Examples of PROs include “How severe is your chest pain?” or “Rate your pain on a scale of 1–10.” PROM data, a subset of PROs, are obtained via psychometrically validated questionnaires that translate patient symptoms and health status into numerical scores. PROM instruments have scoring protocols and unique psychometric characteristics that can be quantified, including content validity, construct validity, and reliability.^10^ Most efforts to quantify PROs in routine clinical practice will employ a PROM instrument as the psychometric properties of these questionnaires have been detailed in validation studies, and therefore this article focuses on the use of PROM data.

## INSTITUTIONAL REVIEW BOARD OVERSIGHT OF PROMS

PROM data have three major use cases: clinical care, QI efforts, and human subjects research. These distinctions are important as IRB oversight is not required for routine clinical care, may be required for QI, and is required for human subjects research.^11^ PROM data often lie at the intersection of the three use cases, and PROMs collected during the course of routine clinical care can be extracted for research and QI purposes alongside other clinical data. For example, a PROM evaluating back pain could be used both during clinical care for symptom monitoring as well as for quality improvement purposes in a spine surgery device registry. Similarly, PROMs assessing heart failure symptoms could be used both to monitor responses to drug therapies as well as for research projects evaluating the association of PROM data with the results of laboratory studies in the clinical record.

The diffuse boundaries between PROM use cases can lead to confusion on the part of clinicians, researchers, administrators, and IRB staff. PROM data are very valuable due to these multiple use cases, and there is potential for PROMs to be collected ostensibly for clinical care (inappropriately bypassing the need for IRB oversight and/or informed consent) when the primary use for these data will be for QI or human subjects research. For example, clinical researchers may seek to deploy a significant number of PROM instruments with a high respondent burden under the auspices of clinical care when the actual intention is to use these data for survey-based research. Similarly, administrators and operational staff may seek to gather more PROM data than can reasonably be justified for use in clinical care with the intention of leveraging this information for value-based health care services. On the other hand, there is potential for IRB staff to inappropriately extend oversight and potentially require consent for PROM data that are collected primarily to inform clinical care. A practical framework for IRB oversight of PROMs can assist IRB staff with the nuance of how these measures can potentially be used in the context of clinical care.

### A practical framework for IRB oversight of PROMs in routine clinical care.

**Figure 2.**
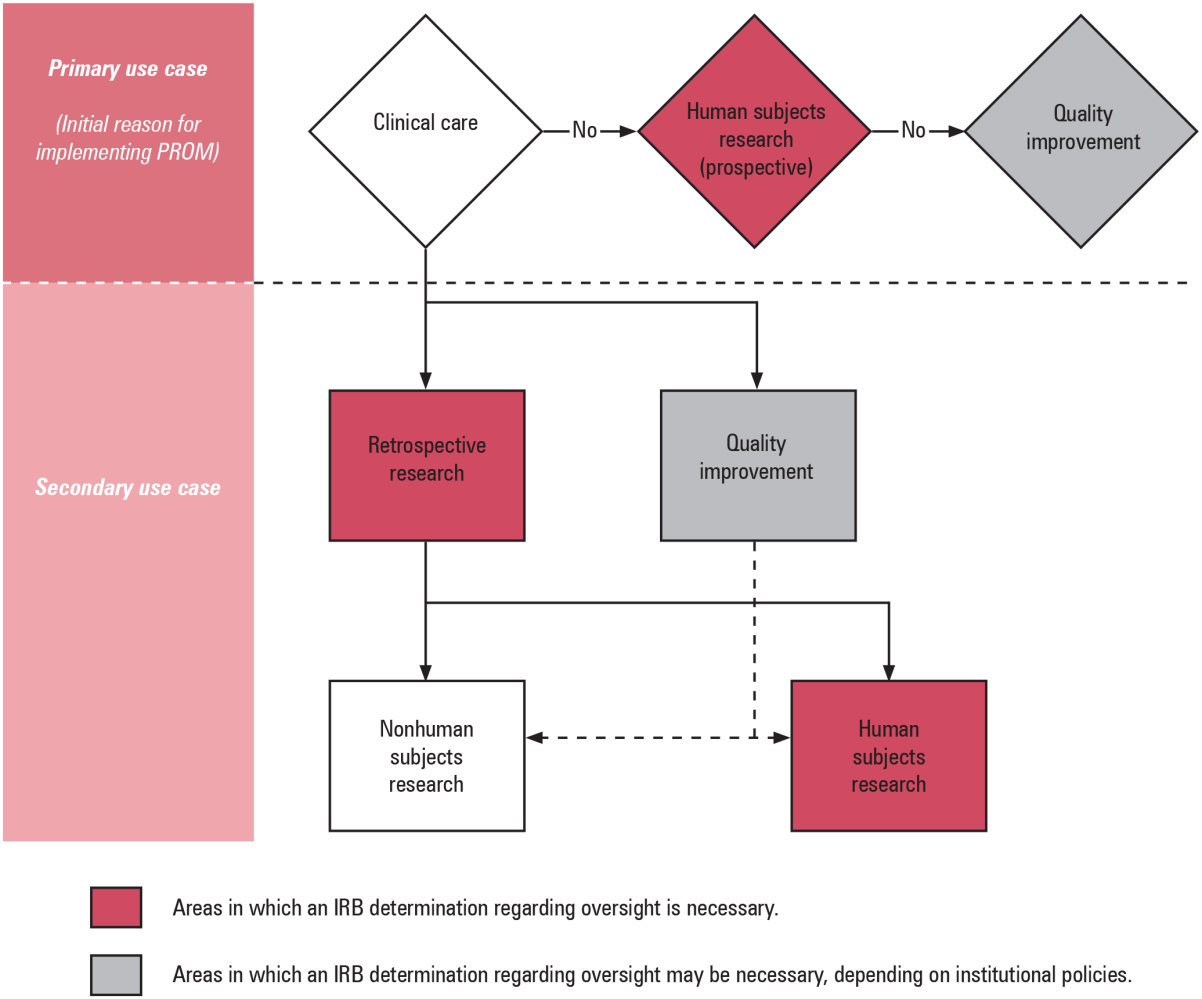
Classifying Use Cases of Patient-Reported Outcome Measures (PROMs) Administered during Routine Clinical Care

For IRB staff, the most important determination is whether the primary purpose of the collected data is to inform clinical care (see figure 2). If so, the PROM collection effort is a part of clinical documentation and does not constitute human subjects research. PROMs that are not implemented primarily for clinical care should be reviewed for a human subjects research determination. PROM data that are not collected primarily for clinical care or human subjects research can generally be classified as QI efforts. Identification of the appropriate primary PROM use case is important as it affects not only the level of IRB oversight required but also risk assessment, whether informed consent is necessary, and whether waivers of consent (or documentation) may be considered for secondary use of data.

### Secondary PROM use cases.

PROM data collected primarily for clinical care can subsequently be used for QI or retrospective research as a secondary use case (see figure 2). From the perspective of IRB oversight, research using PROM data collected primarily to inform routine clinical practice is essentially the same as research involving retrospective extraction of other forms of clinical data, including vital signs and laboratory studies. The secondary use of PROM data can potentially be classified as human subjects research or receive a nonhuman subjects research determination (see figure 2) depending on the nature of the PROM data and other variables extracted alongside these data. Of note, PROMs are often studied using longitudinal data analysis methods and these analyses generally require the date the PROM was completed. Dates are classified as protected health information (PHI) by the Health Insurance Portability and Accountability Act Privacy Rule.^12^ The potential use of PHI is an important part of IRB risk assessment with regard to PROM-related projects.

### Assessing risk in PROMs used in human subjects research.

Most research protocols involving PROMs collected during routine clinical care will involve the secondary use of these data. PROM data extracted from the medical record for secondary use in research are generally minimal risk and eligible for expedited review under Health & Human Services (HHS) Office for Human Research Protections (OHRP) expedited review category 5: “Research involving materials (data, documents, records, or specimens) that have been collected, or will be collected solely for nonresearch purposes (such as medical treatment or diagnosis).”^13^ PROMs extracted from the medical record for secondary use in research could also potentially be categorized as exempt research under exemption category 4 in the revised Common Rule (45 C.F.R. 46.104 d[4][ii]),^14^ which states: “Information, which may include information about biospecimens, is recorded by the investigator in such a manner that the identity of the human subjects cannot readily be ascertained directly or through identifiers linked to the subjects, the investigator does not contact the subjects, and the investigator will not re-identify subjects.” As many PROM-related research projects will involve the use of PHI in the form of dates, these projects can still potentially be categorized as exempt under exemption category 4 provided the data are recorded in a deidentified manner. An example would be recording the number of days between hospital discharge and completion of a PROM in the extracted dataset as opposed to recording the actual dates of discharge and PROM completion.

As discussed further below, research protocols involving PROMs collected primarily for the purpose of human subjects research (i.e., prospective survey-based research) will generally involve primary data collection from questionnaires as opposed to secondary use from an EHR. These projects are usually considered minimal risk and eligible for expedited review under HHS OHRP expedited review category 7: “research employing survey, interview, oral history, focus group, program evaluation, human factors evaluation, or quality assurance methodologies.”^15^ PROMs collected primarily for the purposes of human subjects research could also potentially be categorized as exempt research under exemption category 2 in the revised Common Rule (45 C.F.R. 46.104 d[2]),^16^ provided the IRB determines that there are adequate privacy and confidentiality protections in the study. Of note, exemption category 2 applies to “Research that only includes interactions involving educational tests (cognitive, diagnostic, aptitude, achievement), survey procedures, interview procedures, or observation of public behavior… ,” and thus studies that require additional data elements from the medical record (e.g., laboratory studies, vital signs) may not be exempt. The extent to which exemption category 2 covers activities under expedited review category 7 will likely be dependent on institutional policies and procedures.

### Informed consent.

For nonexempt studies, PROMs collected primarily for human subjects research differ from those collected primarily for clinical care (and later extracted for retrospective research) in eligibility for waivers of consent. Retrospective human subjects research using PROMs collected primarily for clinical care is eligible for a waiver of consent if it meets the five criteria detailed in 45 C.F.R. 46.116(f)(3): (i) minimal risk; (ii) the research cannot practicably be carried out without the waiver; (iii) research involving identifiable private information or biospecimens cannot practicably be carried out without using such information; (iv) the waiver will not adversely affect the rights and welfare of patients; and (v) whenever appropriate, patients will be provided with additional pertinent information after participation. With regard to criterion (ii), it would be impracticable to conduct research on PROM data extracted from large numbers of patients completing these instruments during routine clinical care if retrospective consent were required.

PROM collection efforts conducted primarily for human subjects research (i.e., prospective survey-based research), however, may not be eligible for a waiver of consent as they do not meet criterion (ii) under 46.116(f)(3) above. It is more difficult to justify omitting informed consent for the deployment of survey instruments during routine clinical practice explicitly for the purpose of prospective human subjects research. Exceptions could potentially arise during individual PROM deployment efforts, but most institutions that implement PROMs in routine clinical practice will likely do so at the enterprise level due to the extensive governance and health information technology considerations that these endeavors necessitate.^17^ Accordingly, it is unlikely that informed consent waivers will arise broadly during PROM deployment.

Even when consent is not required in PROM-related projects, efforts should still be made to respect the autonomy and dignity of patients. Morain et al.^18^ proposed several dimensions for demonstrating respect for persons in pragmatic clinical trials that are applicable to research projects involving PROMs collected in routine clinical care. Specifically, the “promoting transparency and open communication” dimension^19^ emphasizes the importance of providing information about research activities and study progress to patients. PROMs used for routine care are often collected by tablets in clinical settings, and clinicians can include introductory screens and text explaining the purpose of administering these questionnaires and their potential to be used for research purposes. Researchers can potentially address this dimension through institutional websites or other materials discussing PROM-related studies. These approaches can also help address the “engaging patients and communities” dimension,^20^ which raises the importance of communicating with patients throughout the lifecycle of research.

### Waivers of documentation.

Though the bar for justifying waivers of consent is much higher for PROM implementations conducted for prospective human subjects research, these efforts could meet the following criteria for a waiver of documentation under 45 C.F.R. 46.117(c)(1): (i) “the only record linking the subject and the research would be the consent document and the principal risk would be potential harm resulting from a breach of confidentiality”; and (ii) “the research presents no more than minimal risk of harm to subjects and involves no procedures for which written consent is normally required outside of the research context.” If granted a waiver of documentation, these PROM collection efforts could still ensure that a consent process encompassing all the required elements of consent occurs and may choose to provide participants with an information sheet explaining the purpose of the research prior to deploying the survey instruments.

Having discussed the differences in IRB oversight and informed consent necessary for PROMs deployed primarily for clinical care as compared to those implemented for prospective human subjects research, we now turn to criteria IRB staff might use to distinguish between these PROM use cases.

## CHARACTERISTICS OF PROMS IMPLEMENTED PRIMARILY FOR ROUTINE CLINICAL CARE

There is a broad rationale for using PROM data to augment clinical care. Specifically, PROMs serve as a clinician-patient communication aid when integrated into routine clinical practice.^21^ A systematic review of the collection of PROMs in oncologic settings found that these measures improved clinician-patient communication, patient satisfaction, and monitoring treatment response when integrated into clinical care.^22^ The effect of implementing PROMs on patient outcomes is less well-described, though there are promising indications that symptom monitoring with PROMs leads to decreased mortality in patients with cancer.^23^ However, PROMs implemented primarily as clinician-patient communication aids should be integrated with clinical processes such that these data can reasonably inform patient care. Clinicians and researchers integrating PROMs into clinical care with no processes through which these data might inform clinical decision-making run the risk of conducting unauthorized human subjects research or quality improvement projects.

We detail several characteristics of PROM implementations that justify clinical care as their primary use case. IRB staff will frequently receive research protocols requesting a nonhuman subjects research determination for primary PROM data collection (due to their deployment for clinical care) and oversight of secondary use of these data for human subjects research. This list will assist IRBs in confirming that the primary PROM data collection does not constitute human subjects research and thus does not require IRB oversight. This list will also help clinicians and researchers ensure that they can justify that PROMs are being used primarily for clinical care, thus facilitating their interactions with IRB staff. Specifically, justifying clinical care as the primary use case of a PROM (see table 1) requires evaluating five major components of the implementation: data platform; survey location; questionnaire length; patient interface; and clinician interface. We discuss each of these in detail.

### Data platform.

PROM implementations require an enabling technology platform. Nearly all institutions using PROMs during routine clinical care will integrate these data into an EHR. PROMs can be collected from patients and displayed to clinicians through the native functionality of the institution’s EHR or via third-party software such as the Research Electronic Data Capture (REDCap) platform.^24^ PROM data that are being used in real time for clinical care should become part of the medical record, alongside other forms of clinical data such as vital signs and laboratory studies. PROM data that are primarily used for research, however, are typically stored in secure databases that are not part of the medical record. IRB staff can use the location of PROM data as an indicator of whether these data are being used in real-time clinical care or for prospective human subjects research.

### Survey location.

Typically, PROMs are deployed to patients in the context of a clinical encounter (i.e., through a patient portal prior to a clinic visit or via a tablet while patients are in the waiting room after clinic check-in). PROMs can also be sent to patients outside a clinical encounter. For example, some patients might receive PROMs through smartphone or web-based portals at specified intervals, for routine symptom monitoring.^25^ PROMs are sometimes used outside of a clinical encounter when routine symptom monitoring can alert clinicians to a deterioration in clinical status. Examples include patients with chronic illnesses such as asthma or heart failure or patients on therapies with significant side effects (such as chemotherapy). In general, the primary use case of PROMs deployed in the context of a clinical encounter is more likely to be for routine clinical care. PROMs deployed outside of a clinical encounter (as with symptom monitoring discussed above) need to be reviewed by a clinician or clinical staff (e.g., sent to their EHR inbox) for the use of these data to be justified as clinical care. Conversely, PROMs that are collected outside of the clinical encounter with no apparatus for a clinician to follow up cannot feasibly be used for clinical care and are in the domain of QI or survey-based human subjects research.

### Questionnaire length.

IRB staff should assess the respondent burden, or length of time necessary for patients to complete the PROM questionnaires. PROMs deployed in real-time clinical practice are typically concise and efficient with minimal disruption of the clinical workflow. There are no guidelines on the specific amount of time or number of questions recommended for PROM implementations in routine clinical care. Large batteries of questions, however, can potentially lead to respondent fatigue and are the domain of survey-based research.^26^ Our own experience suggests that questionnaires should generally be kept under 30 items to avoid respondent fatigue and the potential to slow clinic check-in processes. However, there are notable exceptions to this rule. Some clinical specialties may routinely employ questionnaires that are both considerably longer than the norm and necessary for the clinic’s practice. This is often the case for specialties requiring extensive use of behavioral, developmental, and neurological assessments (e.g., pediatric developmental medicine).

### Patient interface.

As with the data platform above, PROM instruments collected primarily for clinical care are generally integrated into the EHR as well as the clinical workflow and thus presented seamlessly to patients. At our institution, PROMs are collected with tablets as part of our paperless clinic processes. In this manner, PROM questions are asked alongside patient history items during clinic check-in. PROMs that are deployed to patients using a modality separate from the EHR, such as paper surveys, are less likely to be used for clinical care. Of note, some EHRs require third-party integrations or tools to administer PROMs due to the complexity of the questionnaires (e.g., branching logic, complex scoring protocols). PROM implementations using third-party integrations may need to be reviewed on a case-by-case basis.

### Clinician interface.

IRB staff should also assess whether the collected PROM data could reasonably inform clinical care. PROMs that best inform clinical care are highly interpretable, have well-defined scoring protocols, and can potentially lead to changes in management.^27^ The Patient Health Questionnaire-9 (PHQ-9),^28^ for example, has cut points for mild, moderate, and severe depression, assisting in treatment. The Asthma Control Test^29^ characterizes the severity of asthma symptoms and can be used to guide therapies such as corticosteroids. PROM implementations that would be more appropriately classified as QI or survey-based research, on the other hand, collect data that may not necessarily change clinical management. Additionally, PROMs can only inform clinical care if they are collected and displayed to clinicians in real-time. Doing so requires an enabling technology platform, ideally an EHR that can collect these data and display them to clinicians with appropriate visualizations and tables in clinical notes if necessary. Questionnaire data that are returned to clinicians with a significant amount of latency (e.g., monthly or quarterly reports), however, cannot be expected to inform clinical care.

Having discussed the characteristics of PROMs used primarily for clinical care, we now focus specifically on IRB oversight of PROM data used for QI purposes.

## PROMS AND QUALITY IMPROVEMENT

IRB oversight of QI efforts is an extensive topic, and a detailed review of the subject would be outside the scope of this article. Our illustration of PROM QI use cases is guided by the framework of the 2006 *Hastings Center Report* on QI methods,^30^ which distinguished between nonresearch QI involving human data sources (exempt from IRB oversight) and nonexempt QI that is also human subjects research (QI/HuSR). However, we recognize that the boundaries between exempt QI and QI/HuSR can be diffuse. For example, Finkelstein et al.^31^ characterized QI activities as routine (activities to improve health care processes), nonroutine (activities to both improve health care processes and contribute to generalized knowledge), or QI research (designing and testing new methods to improve health care quality, producing generalizable knowledge). This group deemed that nonroutine QI requires oversight, though not necessarily by an IRB. Given the fluid boundaries between QI activities, IRB QI oversight is variable between institutions and thus dependent on institutional policies and procedures. PROM data can be used for QI activities in two major ways: as predictors of relevant outcomes, such as mortality and readmissions; and as the outcome of interest themselves. We discuss these two forms of QI use cases to provide context for potential IRB oversight regarding them.

### PROMs as predictors.

Many PROM instruments are highly sensitive to changes in health status and predictive of clinical outcomes. For example, both baseline Kansas City Cardiomyopathy Score Short Form (KCCQ-12) scores and changes in these scores over time are highly predictive of mortality and readmissions.^32^ As continuous variables, PROMs can easily be integrated into various forms of predictive models. These models can be used to create dashboards that QI staff can use to identify patients at risk. IRB determinations regarding whether a project constitutes exempt QI versus QI/HuSR can vary according to how well the predictive capabilities of the project’s PROM instruments are validated. For example, initial analyses to characterize the KCCQ-12’s ability to predict hospitalizations in a segment of outpatients would likely be categorized as nonexempt QI/HuSR. A project to use data from these initial analyses to incorporate the KCCQ-12 into institutional processes for minimizing hospitalizations in high-risk patients (e.g. follow-up calls from care coordinators) would most likely be characterized as exempt QI.

### PROMs as the outcome of interest.

As measures of outcomes that are relevant to patients, PROMs can serve as the outcome of interest in and of themselves. QI staff can use PROM data to monitor the health status of various patient populations within an institution. This PROM use case is particularly relevant to QI staff interested in health equity,^33^ as PROMs can serve as a lens for evaluating disparities in outcomes across geography, race, ethnicity, age, and a host of other socioeconomic factors. Payers are also increasingly interested in PROMs, combining these data with costs as a measure of health care value.^34^ For example, relative improvement in health status (as defined by disease-specific PROMs) could be quantified before and after surgical procedures using PROM instruments. Projects that utilize these outcome data for bundles and value-based health care contracts would most likely be characterized as exempt QI. Some projects evaluate outcomes before and after procedures with the purpose of creating generalizable knowledge, however. For example, a project utilizing PROMs collected during routine clinical practice to evaluate the comparative effectiveness of surgery and radiation therapy for prostate cancer would most likely constitute QI/HuSR and thus potentially require IRB oversight.

## CONCLUSION

The use of PROMs in routine clinical practice spans multiple use cases including clinical care, human subjects research, and QI. The fluid boundaries between use cases for PROM data can lead to confusion among IRB staff concerning where to apply oversight. These difficulties are heightened by the diffuse boundaries between QI and QI/HuSR. In this article we have sought to provide practical guidance for IRBs in evaluating the level of oversight required during PROM implementations. IRB determinations regarding these activities are likely to vary somewhat among institutions. However, a pragmatic understanding of the various PROM use cases will assist IRB staff and clinicians who seek to better measure health status and the value of care provided to patients.

## Figures and Tables

**Table 1.** Criteria for Characterizing the Primary Use Case of PROM Implementations as Clinical Care

PROM components	Criteria (yes/no)
Data platform	Are PROM data part of the medical record, alongside other clinical data such as vital signs and laboratory studies?
Survey location	Are PROM questionnaires deployed in the context of a clinical encounter? Are PROMs completed by patients outside of the clinical encounter (e.g. at home via a smartphone) sent to clinicians or their staff for review?
Questionnaire length	Do the PROM instruments involve a reasonable respondent burden (i.e., time necessary to complete the questionnaires)?
Patient interface	Is the patient-facing interface used to collect PROMs fully integrated with the EHR system?
Clinician interface	Can the collected PROMs potentially change clinical management? Are PROM data displayed to clinicians in real time, as with other clinical results?
